# Doctors’ Perceptions, Attitudes and Practices towards the Management of Multidrug-Resistant Organism Infections after the Implementation of an Antimicrobial Stewardship Programme during the COVID-19 Pandemic

**DOI:** 10.3390/tropicalmed6010020

**Published:** 2021-02-05

**Authors:** Nikolaos Spernovasilis, Despo Ierodiakonou, Christos Spanias, Anna Mathioudaki, Petros Ioannou, Emmanouil C. Petrakis, Diamantis P. Kofteridis

**Affiliations:** 1 School of Medicine, University of Crete, 71003 Heraklion, Greece; nikspe@hotmail.com; 2 Department of Social Medicine, School of Medicine, University of Crete, 71003 Heraklion, Greece; desierod@gmail.com; 3 Department of Primary Care and Population Health, University of Nicosia Medical School, Nicosia 2417, Cyprus; 4 Department of Pharmacy, University Hospital of Heraklion, 71110 Heraklion, Greece; chspanias@pagni.gr; 5 Department of Internal Medicine, University Hospital of Heraklion, 71110 Heraklion, Greece; mathiouanna94@gmail.com (A.M.); petros_io@hotmail.com (P.I.); mpetrakis86@gmail.com (E.C.P.)

**Keywords:** stewardship, antimicrobial use, antimicrobial resistance, perceptions, attitudes, practices, infection control, COVID-19

## Abstract

Background: Greece is among the European countries with the highest consumption of antibiotics, both in community and hospital settings, including last-line antibiotics, such as carbapenems. We sought to explore doctors’ perceptions, attitudes and practices towards the management of patients with multidrug-resistant organism (MDRO) infections after the implementation of an antimicrobial stewardship programme (ASP) in a tertiary academic hospital during the COVID-19 pandemic. Methods: A self-administered, internet-based questionnaire survey was completed by doctors of the University Hospital of Heraklion in Crete, Greece. Results: In total, 202 (59.1%) hospital doctors fully completed the questionnaire. Most of them agreed that the prospective audit and feedback ASP strategy is more effective and educational than the preauthorization ASP strategy. ASP implementation prompted most respondents to monitor the continuously evolving microbiological data of their patients more closely and affected them towards a multidisciplinary and personalised care of patients with infections caused by MDROs and towards a more rigorous implementation of infection prevention and control measures. The vast majority of participants (98.5%) stated that ASP must be continued and further developed during the COVID-19 pandemic. Conclusion: The ASP implementation in our hospital had a beneficial impact on doctors’ perceptions, attitudes and practices with regard to the management of infections due to MDROs.

## 1. Introduction

Excessive antimicrobial consumption and misuse are major problems worldwide and significantly contribute to antimicrobial resistance [1]. The emergence and spread of antimicrobial resistance negatively affect patient outcomes, healthcare costs and the enduring efficacy of antimicrobial agents [2,3]. Antibiotic consumption in Greece ranks among the highest in Europe, both in the community and the hospital sector [4], whereas recent data for other categories of antimicrobials do not exist. In parallel, antimicrobial resistance (AMR) rates in Greece are extremely high during this decade [5]. Therefore, a national action plan on AMR is currently under development [6], while many Greek hospitals have already optimised infection prevention and control (IPC) practices and implemented antimicrobial stewardship programmes (ASPs).

Since the beginning of 2020, an ASP has been implemented for a first time in the adult clinics of the University Hospital of Heraklion in Greece. This ASP is focused on the prescription of carbapenems with regard to the indication, dosage and duration of treatment, combined with the judicious use of carbapenem-sparing antibiotics whenever appropriate. The programme is based on the prospective audit and feedback strategy, along with a case-based education of treating doctors. An infectious diseases (ID) specialist and an ID fellow are being alerted by the hospital pharmacy upon prescription request for carbapenem and provide unsolicited in-person (“handshake”) consultation within 72 h for all patients for whom the treating doctors have prescribed carbapenem. This approach includes a lack of prior authorization by the ASP members for carbapenem administration (i.e., treating doctors can prescribe a carbapenem for their patients without previous approval and even continue carbapenem administration despite a potentially opposite recommendation by the ASP members), the patient’s clinical examination by the ID specialist or ID fellow, review of the patient’s laboratory data and of all prescribed antimicrobials, and a subsequent daily, rounding-based, in-person approach to feedback by the ID doctors. Further ID consultation service upon request is available 7 days a week, 24 h a day, through telephone or in-person. The execution of the ASP has not been affected by the COVID-19 pandemic, since our hospital’s capacity has not been exceeded during the care of COVID-19 patients. In this context, and after eleven months of ASP implementation, we sought to examine doctors’ perceptions, attitudes and practices towards the management of patients with multidrug-resistant organism (MDRO) infections. To the best of our knowledge, this was the first study of its kind conducted during the COVID-19 pandemic.

## 2. Materials and Methods

### 2.1. Study Design, Setting, Duration and Participants

A cross-sectional web-based survey was conducted from 21 November to 4 December 2020 at the 760-bed University Hospital of Heraklion in Greece. All resident and specialist doctors of hospital adult clinics were eligible to participate.

### 2.2. Survey Instrument

A self-administered questionnaire was developed on the SurveyMonkey platform (SurveyMonkey Inc., San Mateo, CA, USA) by a multidisciplinary team of infectious diseases specialists and fellows, clinical pharmacists and hospital epidemiologists. It was partially based on previously validated questionnaires in the published literature [7,8]. It consisted of 15 items, including close-ended, multiple choice and Likert-scale questions (with sub-questions), divided as follows: 5 on demographics and practice-related information; 4 on previous and current experience with ASP; 4 on perceptions related to the management of patients with MDRO infections after ASP implementation; and 2 on attitudes and practices towards the management of patients’ MDRO infections after ASP implementation. The questionnaire is available as Supplementary Material. Prior to dissemination, the questionnaire was piloted among 10 resident and specialist doctors to assess length and readability.

### 2.3. Participation and Ethical Approval

Participation was voluntary, anonymous and without compensation. The invitation to participate was sent via email through the SurveyMonkey platform. Questionnaires not completely answered within 10 days generated a single reminder email. Informed consent for the questionnaire’s completion was declared on its first page. This study was approved by the hospital’s Ethics Committee.

### 2.4. Statistical Analysis

Data coding and descriptive statistical analyses were performed in R version 3.6.2 (12 December 2019) (R Foundation for Statistical Computing, Vienna, Austria). Qualitative data are presented as counts and percentages. Continuous variables were assessed for normality and due to not normal distributions are presented as median and interquartile range. In addition, we used chi-square, Fisher’s exact and Mann–Whitney U tests for assessing the differences according to the level of practice (resident versus specialist doctor) and to specialty (medical versus surgical). Significance level was set at 5%.

## 3. Results

### 3.1. Participants

Three hundred and forty-two hospital doctors were eligible to participate in this study. A total of 202 (59.1%) responded with the full completion of the questionnaire and were included in the analysis. Among them, 105 (52%) were residents and 97 (48%) were specialists. There was representation from all hospital adult specialties. Table 1 shows the basic characteristics of the respondents and their experience with previous and current ASPs.

### 3.2. Perceptions

**Figure 1:**
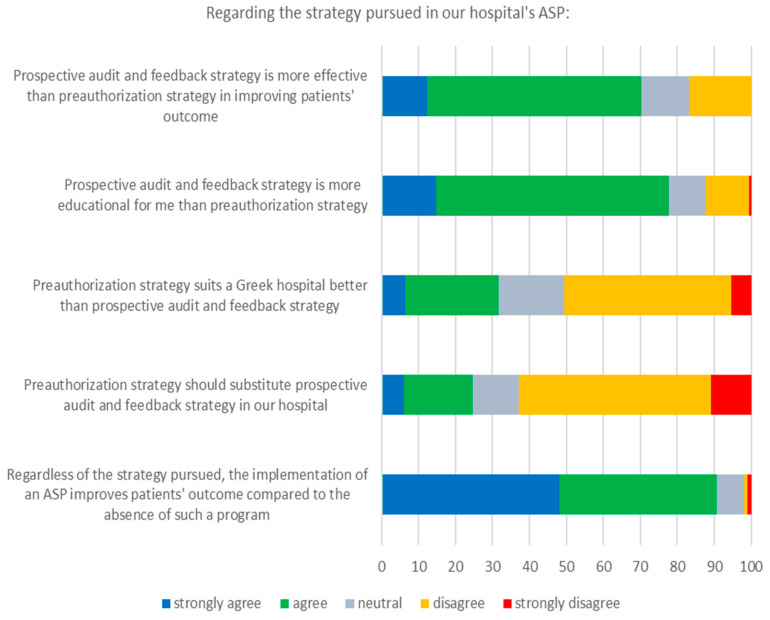
Respondents’ perceptions regarding the strategy pursued in our hospital’s ASP.

Respondents’ perceptions in relation to the pursued ASP strategy are presented in Figure 1. The great majority of doctors believed that the prospective audit and feedback ASP strategy is more effective and educational than the preauthorization ASP strategy (70.3% and 77.7%, respectively). Most respondents (90.6%) agreed that the implementation of an ASP improves the patients’ outcome compared to the absence of such a programme regardless of the pursued strategy, even though a third of participants considered that the preauthorization strategy suits a Greek hospital better; however, less than 25% of participants agreed that the prospective audit and feedback strategy of the current ASP should change.

**Figure 2:**
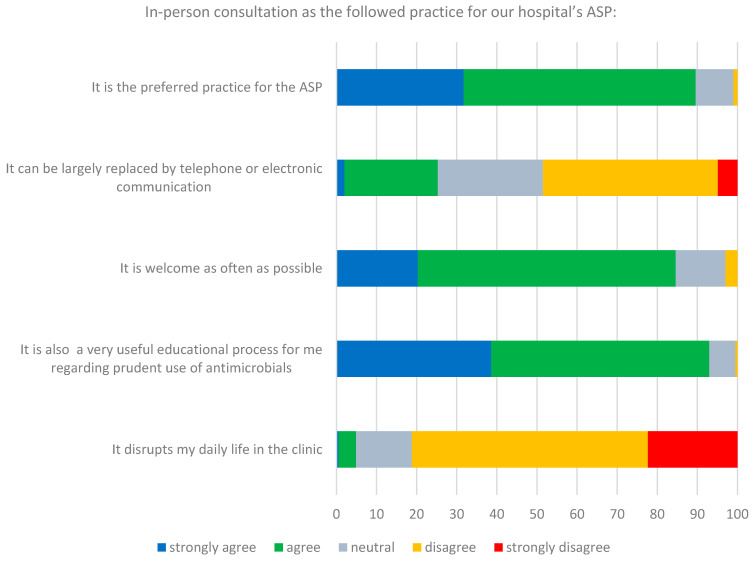
Respondents’ perceptions regarding in-person consultation as the followed practice for our hospital’s ASP.

More than 80% of respondents agreed that in-person consultation is the preferred practice for the ASP, welcome as often as possible, constituting at the same time an educational process for the treating doctors (Figure 2). Only 5% of respondents thought that in-person consultation disrupts their daily clinical practice, while approximately one-fourth of participants reported that it can be largely replaced by telephone or electronic communication.

**Figure 3:**
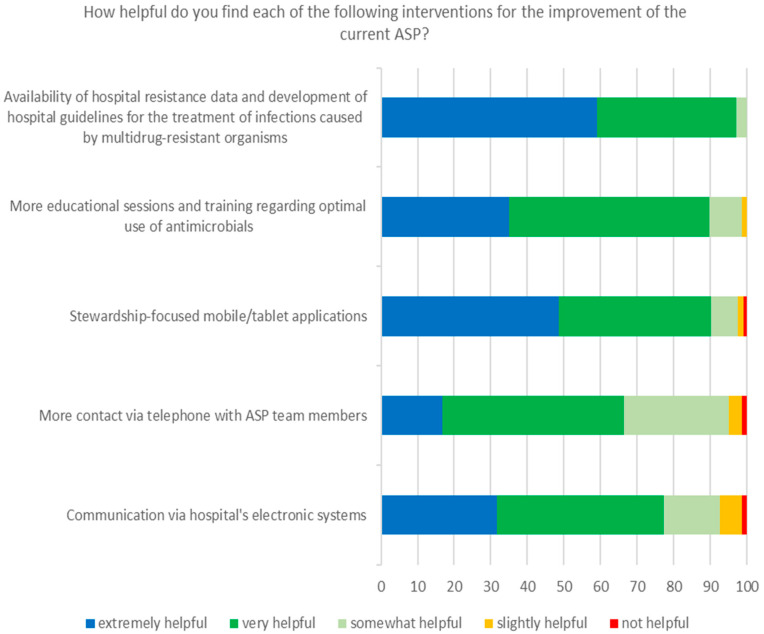
Respondents’ perceptions regarding potential interventions to improve our hospital’s ASP.

Regarding the proposed measures for the improvement of the current ASP (Figure 3), the majority of participants agreed that these could be helpful, with the most popular preferences in the following order: availability of hospital resistance data and the development of hospital guidelines for the treatment of infections caused by MDROs, more educational sessions and training regarding the optimal use of antimicrobials, and the availability of stewardship-focused mobile/tablet applications. Noteworthy, in a subsequent question regarding the future of the ASP in our hospital during the COVID-19 pandemic, 98.5% of respondents stated that ASP must be continued and further developed, and only 1.5% that it must be postponed.

### 3.3. Attitudes

**Figure 4:**
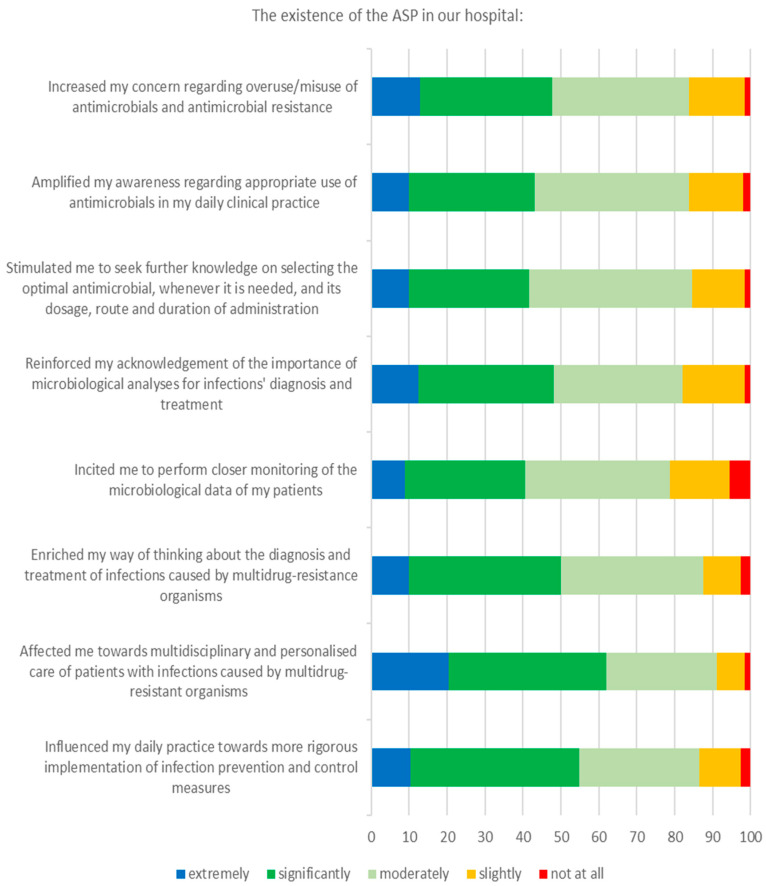
Respondents’ attitudes and practices towards the management of patients with MDRO infections.

The impact of the ASP implementation on respondents’ attitudes regarding the management of patients with MDRO infections seemed to be quite positive (Figure 4). Specifically, ASP existence increased, at least moderately, most doctors’ (>80%) concern regarding the overuse/misuse of antimicrobials and antimicrobial resistance, and amplified their awareness regarding the appropriate use of antimicrobials in their daily clinical practice. Similarly, ASP reinforced their acknowledgement of the importance of microbiological analyses and enriched their way of thinking about the diagnosis and treatment of infections caused by MDROs. In a separate question regarding respondents’ willingness to participate more actively in the ASP in the future, 67.3% responded positively and 33.7% negatively.

### 3.4. Practices

ASP incited the majority of respondents (>80%) to perform closer monitoring of the microbiological data of their patients and stimulated them to seek further knowledge on selecting the optimal antimicrobial treatment for patients with infections caused by MDROs (Figure 4). In addition, ASP had a beneficial impact on most respondents (>85%) towards a multidisciplinary and personalised care of patients and towards a more rigorous implementation of IPC measures. Notably, with regard to respondents’ perceptions, attitudes and practices, no statistically significant differences were identified between residents and specialists, and between medical and surgical specialties (data not shown).

## 4. Discussion

This study was the first to examine the perceptions, attitudes and practices of hospital doctors towards the management of hospitalised patients with infections caused by MDROs after the implementation of an ASP during the COVID-19 pandemic. The study’s significance lies not only on its actual findings, but also in the fact that it was conducted under the pressure that COVID-19 put on health systems and healthcare workers worldwide.

The response rate of invited doctors in the current survey (59.1%) was comparable to or higher than most similar studies [7,8,9,10,11]. Both genders were represented almost equally in the study sample, as was also the case regarding the professional status, i.e., resident or specialist. About half of respondents reported that they always accept ASP team recommendations and about a third reported that they often do, in line with the adherence rates of 68–81% that have been reported in the literature [12].

The majority of participants in this study perceived the prospective audit and feedback strategy as more educational for the prescribers and more effective for patients’ favourable outcome compared with the preauthorization strategy. Furthermore, the existence of an ASP was perceived as a contributing factor for improved patients’ outcome compared with its absence. Indeed, the prospective audit and feedback strategy represents a more educational process for the prescribers through evidence-based discussions between them and the ASP team members [13]. However, no rigorously designed studies directly compare prospective audit and feedback to preauthorization with respect to patient outcomes [14]. In addition, current literature data show that ASPs, regardless of the strategy pursued, reduce patients’ duration of treatment and hospital stay, but they do not affect mortality [15]. Thus, some of the aforementioned perceptions may simply reflect the fact that doctors do not favour interventions that limit their prescribing autonomy.

Most respondents agreed that in-person consultation is the preferred practice for our hospital’s ASP, however, the majority of respondents did not have previous experience with ASPs, making this positive perception of in-person consultation less objective. Regardless of that, in-person consultation was perceived as an educational interaction which is desirable as often as possible. The latter, in combination with what was mentioned in the preceding paragraph, further highlights doctors’ tendency for additional education on antimicrobial prescribing in a country characterised by inappropriate use of antimicrobials and high resistance rates [16]. This tendency is confirmed by the fact that the demand for more educational sessions and training regarding the optimal use of antimicrobials was among the most popular interventions that participants want to be included or enhanced in the current ASP, along with the availability of hospital resistance data and guidelines.

A notable finding of the present study is that the vast majority of respondents wanted the ASP to be continued and further developed despite the fact that their workload had already been increased due to the COVID-19 pandemic [17]. This is quite encouraging, since high and inappropriate antimicrobial use has been observed during this pandemic [18,19]. Many studies revealed heavy use of empirical broad-spectrum antimicrobials in hospitals while evidence so far suggests that the rates of bacterial and fungal infection in COVID-19 patients are rather low [20,21]. Therefore, the integration of an ASP in every hospital’s COVID-19 response effort is imperative.

The ASP implementation in our hospital had a beneficial impact on doctors’ attitudes regarding the management of infections due to MDROs. Most of the participants reported an increase in their concern about the imprudent use of antimicrobials and in their awareness on this issue. They also reported a greater recognition of the importance of microbiological analyses, including Gram stain, cultures, molecular techniques and serology, which is a prerequisite for a successful ASP [22], and enrichment of their approach to managing MDRO infections. Interestingly, about two-thirds of respondents would be willing to participate in ASP activities to improve the quality of antimicrobial use in the hospital, a proportion similar to or even higher than that observed in other studies [7,8,23].

One of the most important findings of this study was the observed change in doctors’ practices in their daily clinical activity, eleven months after ASP implementation. In particular, ASP implementation prompted most respondents to more closely monitor the continuously evolving microbiological data of their patients. Furthermore, respondents were affected towards a multidisciplinary and personalised care of patients with infections caused by MDROs, which is essential for a favourable outcome in many cases, especially during the COVID-19 pandemic [24,25]. In addition, a more rigorous implementation of IPC measures after the ASP initiation was reported by the majority of respondents, an encouraging finding considering that a successful ASP concurrently requires well-performing IPC practices [26].

The ASP of our hospital will incorporate the potential interventions that the participants of this study found most helpful, such as the development of hospital guidelines for the treatment of MDRO infections, more educational sessions regarding the prudent administration of antimicrobials, and the use of stewardship-focused mobile or tablet applications. Moreover, in the near future, we will examine the impact of the ASP on patients’ outcomes, on hospital antibiotic consumption and on hospital AMR, by comparing pre- and post-ASP implementation periods. Finally, the ASP will be expanded in order to monitor and direct the appropriate use of additional antimicrobials.

This study has certain limitations that should be mentioned. The survey was conducted at a single site, a well-resourced academic hospital whose capacity has not been exceeded during COVID-19 pandemic, therefore the results should be generalised with caution. In addition, as a survey, responses are prone to social desirability bias; confidentiality minimised this as much as possible. Furthermore, participation was voluntary, and volunteer bias is possible; however, the response rate was relatively high and all targeted departments were represented, therefore, there is confidence that the results are representative of the study population.

## 5. Conclusions

This study demonstrates a positive impact of ASP implementation on hospital doctors’ perceptions, attitudes and practices towards the management of patients with MDRO infections. The study also confirms that doctors find the continuation of ASPs during the COVID-19 pandemic supportive and beneficial. The findings of this study will be useful for the design, implementation and further development of hospital ASPs.

## Figures and Tables

**Table 1:** Characteristics of the survey respondents and their experience with ASPs.

Characteristic *	Total (n = 202)	Residents (n = 105)	Specialists (n = 97)
Age, median (IQR)	37 (30–46)	30 (27–33)	46 (40–53)
Gender			
Male	114 (56.4)	50 (47.6)	64 (66)
Female	88 (43.6)	55 (52.4)	33 (34)
Specialty			
Medical	124 (61.4)	76 (72.4)	48 (49.5)
Surgical	65 (32.2)	29 (27.6)	36 (37.1)
ICU	13 (6.4)	0 (0)	13 (13.4)
Years of experience, median (IQR)			
In residency	n/a	4 (2–5)	n/a
Post-residency	n/a	n/a	11 (5–19)
Previous experience with ASPs			
Yes	29 (14.4)	9 (8.6)	20 (20.6)
No	173 (85.6)	96 (91.4)	77 (79.4)
Rate of patients with MDR Gram-negative infection under respondents’ care			
Zero	23 (11.4)	14 (13.3)	9 (9.3)
1–4 cases/month	120 (59.4)	72 (68.6)	48 (49.5)
5–10 cases/month	34 (16.8)	13 (12.4)	21 (21.6)
>10 cases/month	25 (12.4)	6 (5.7)	19 (19.6)
Rate of ASP consultation for patients with MDR Gram-negative infection under respondents’ care			
Zero	29 (14.3)	20 (19)	9 (9.3)
1–4 times/month	125 (61.9)	70 (66.7)	55 (56.7)
5–10 times/month	40 (19.8)	13 (12.4)	27 (27.8)
>10 times/month	8 (4)	2 (1.9)	6 (6.2)
Respondents’ adherence to ASP team recommendations			
Never	5 (2.5)	5 (4.8)	0 (0)
Rarely	5 (2.5)	1 (0.9)	4 (4.1)
Sometimes	32 (15.8)	14 (13.3)	18 (18.6)
Often	67 (33.2)	34 (32.4)	33 (34)
Always	93 (46)	51 (48.6)	42 (43.3)

* All data are in n (%), unless otherwise indicated. ASPs: antimicrobial stewardship programmes; IQR: interquartile range; ICU: intensive care unit; n/a: not available; MDR: multidrug-resistant.

## Data Availability

The data presented in this study are available on request from the corresponding author.

## Supplementary Materials

The following are available online at https://www.mdpi.com/2414-6366/6/1/20/s1. Questionnaire.
