# Supplementary material for: Doctors’ Perceptions, Attitudes and Practices towards the Management of Multidrug-Resistant Organism Infections after the Implementation of an Antimicrobial Stewardship Programme during the COVID-19 Pandemic
Source: Trop Med Infect Dis. 2021 Feb 5;6(1):20. doi: 10.3390/tropicalmed6010020 (PMC7930958; doi:10.3390/tropicalmed6010020)
Supplement: Supplementary file 1 [file tropicalmed-06-00020-s001.pdf]

Questionnaire

General information

1. Age: ☐

2. Gender:

Male ☐
Female ☐

3. What is your professional status?

Resident doctor ☐
Specialist doctor ☐

4. What is your specialty;

Vascular surgery ☐	Haematology ☐	Gastroenterology ☐
General practice ☐	General surgery ☐	Dermatology ☐
Endocrinology ☐	Cardiology ☐	Cardiac surgery ☐
Neurology ☐	Neurosurgery ☐	Nephrology ☐
Orthopaedic ☐	Urology ☐	Ophthalmology ☐
Internal medicine ☐	Oncology ☐	Obstetrics & Gynaecology ☐
Plastic surgery ☐	Respiratory medicine ☐	Rheumatology ☐
Psychiatry ☐	ENT ☐	Thoracic surgery ☐
ICU ☐	Craniofacial surgery ☐	

5. How many years of experience do you have as a doctor? (If you are a specialist doctor, please report only the years of experience you have as a specialist doctor) ☐

6. Do you have previous experience (>3 months) with antimicrobial stewardship programmes (ASPs)?

Yes ☐ No ☐

7. How often do you have patients with multidrug-resistant (MDR) Gram-negative infections under your care?

Not at all ☐
1-4 times/month ☐
5-10 times/month ☐
>10 times/month ☐

8. How often do you seek ASP consultation for patients with MDR Gram-negative infections who are under your care?

Not at all ☐
1-4 times/month ☐
5-10 times/month ☐
>10 times/month ☐

9. How often do you accept the recommendations provided by the ASP team?

Never ☐
Rarely ☐
Sometimes ☐
Often ☐
Always ☐

Perceptions

10. How much do you disagree or agree with each of the following statements?

1 = Strongly disagree, 2 = Disagree, 3 = Neutral, 4 = Agree, 5 = Strongly agree

- Prospective audit and feedback strategy is more effective than preauthorization strategy in improving patients' outcome
  ☐1 ☐ 2 ☐ 3 ☐4 ☐5

- Prospective audit and feedback strategy is more educational for me than preauthorization strategy
  ☐1 ☐ 2 ☐ 3 ☐4 ☐5

- Preauthorization strategy suits a Greek hospital better than prospective audit and feedback strategy
  ☐1 ☐ 2 ☐ 3 ☐4 ☐5

- Preauthorization strategy should substitute prospective audit and feedback strategy in our hospital
  ☐1 ☐ 2 ☐ 3 ☐4 ☐5

- Regardless of the strategy followed, the implementation of an ASP improves patients' outcome compared to the absence of such a program
  ☐1 ☐ 2 ☐ 3 ☐4 ☐5

11. How much do you disagree or agree with each of the following statements regarding in-person consultation as the followed practice for the ASP in our hospital?

1 = Strongly disagree, 2 = Disagree, 3 = Neutral, 4 = Agree, 5 = Strongly agree

- It is the preferred practice for the ASP
  ☐1 ☐ 2 ☐ 3 ☐4 ☐5

- It can be largely replaced by telephone or electronic communication

☐1 ☐ 2 ☐ 3 ☐4 ☐5

- It is welcome as often as possible
  ☐1 ☐ 2 ☐ 3 ☐4 ☐5

- It is also a very useful educational process for me regarding prudent use of antimicrobials
  ☐1 ☐ 2 ☐ 3 ☐4 ☐5

- It disrupts my daily life in the clinic
  ☐1 ☐ 2 ☐ 3 ☐4 ☐5

12. How helpful do you find each of the following interventions for the improvement of the current ASP?

1 = Not helpful, 2 = Slightly helpful, 3 = Somewhat helpful, 4 = Very helpful, 5 = Extremely helpful

- Availability of hospital resistance data and development of hospital guidelines for the treatment of infections caused by multidrug-resistant organisms
  ☐1 ☐ 2 ☐ 3 ☐4 ☐5

- More educational sessions and training regarding optimal use of antimicrobials
  ☐1 ☐ 2 ☐ 3 ☐4 ☐5

- Stewardship-focused mobile/tablet applications
  ☐1 ☐ 2 ☐ 3 ☐4 ☐5

- More contact via telephone with ASP team members
  ☐1 ☐ 2 ☐ 3 ☐4 ☐5

- Communication via hospital's electronic systems
  ☐1 ☐ 2 ☐ 3 ☐4 ☐5

13. During COVID-19 pandemic, the ASP must be:

continued and further developed ☐
postponed ☐

Attitudes and Practices

14. The existence of the ASP in our hospital:

1 = Not at all, 2 = Slightly, 3 = Moderately, 4 = Significantly, 5 = Extremely

- Increased my concern regarding overuse/misuse of antimicrobials and antimicrobial resistance
  ☐1 ☐ 2 ☐ 3 ☐4 ☐5

- Amplified my awareness regarding appropriate use of antimicrobials in my daily clinical practice
  ☐1 ☐ 2 ☐ 3 ☐4 ☐5

- Stimulated me to seek further knowledge on selecting the optimal antimicrobial, whenever it is needed, and its dosage, route and duration of administration
  ☐1 ☐ 2 ☐ 3 ☐4 ☐5

- Reinforced my acknowledgement of the importance of microbiological analyses for infections' diagnosis and treatment
  ☐1 ☐ 2 ☐ 3 ☐4 ☐5

- Incited me to perform closer monitoring of the microbiological data of my patients
  ☐1 ☐ 2 ☐ 3 ☐4 ☐5

- Enriched my way of thinking about the diagnosis and treatment of infections caused by multidrug-resistance organisms

☐1 ☐ 2 ☐ 3 ☐4 ☐5

- Affected me towards multidisciplinary and personalised care of patients with infections caused by multidrug-resistant organisms
  ☐1 ☐ 2 ☐ 3 ☐4 ☐5

- Influenced my daily practice towards more rigorous implementation of infection prevention and control measures
  ☐1 ☐ 2 ☐ 3 ☐4 ☐5

15. Would you be willing to participate more actively in the ASP in the future?

Yes ☐ No ☐
